# Impact of Lack of Breast Feeding during Neonatal Age on the Development of Clinical Signs of Pneumonia and Hypoxemia in Young Infants with Diarrhea

**DOI:** 10.1371/journal.pone.0025817

**Published:** 2011-10-03

**Authors:** Mohammod J. Chisti, Mohammed A. Salam, Jonathan Harvey Smith, Tahmeed Ahmed, Hasan Ashraf, Pradip K. Bardhan, Mark A. C. Pietroni

**Affiliations:** 1 Clinical Sciences Division, International Centre for Diarrhoeal Disease Research Bangladesh, Mohakhali, Dhaka, Bangladesh; 2 Executive Directors Division, International Centre for Diarrhoeal Disease Research Bangladesh, Mohakhali, Dhaka, Bangladesh; 3 Portex Department of Paediatric Anaesthesia, Institute of Child Health, University College London, London, United Kingdom; 4 Nutrition Programme, Clinical Sciences Division, International Centre for Diarrhoeal Disease Research Bangladesh, Mohakhali, Dhaka, Bangladesh; Hôpital Robert Debré, France

## Abstract

**Background:**

Hypoxemia is a grave sequel of pneumonia, and an important predictor of a fatal outcome. Pneumonia in the neonatal period is often associated with lack of breast feeding. However, there is no published report on the impact of the cessation of breast feeding in the neonatal period on the development of pneumonia and hypoxemia. The purpose of our study was to assess the impact of non-breast feeding or stopping breast feeding during the neonatal period (henceforth to be referred to as non-breast fed) on clinical features of pneumonia and hypoxemia in 0–6-month-old infants with diarrhea admitted to an urban hospital in Bangladesh.

**Methods:**

We prospectively enrolled all infants (n = 107) aged 0 to 6 months who were admitted to the Special Care Ward (SCW) of the Dhaka Hospital of the International Centre for Diarrhoeal Disease Research Bangladesh (ICDDR,B) with diarrhea and pneumonia from September 2007 through December 2007.We compared the clinical features of pneumonia and hypoxemia of breast fed infants (n = 34) with those who were non-breast fed (n = 73).

**Results:**

The median (inter-quartile range) duration of hypoxemia (hours) in non-breast-feds was longer than breast-fed infants [0.0 (0.0, 12.0) vs. 12.0 (0.0, 21.75); p = 0.021]. After adjusting for potential confounders such as inability to drink, fever, head nodding, cyanosis, grunting respiration, and lower chest wall in drawing, the non-breast-fed infants with pneumonia along with diarrhea had a higher probability of cough (OR 9.09; CI 1.34–61.71; p = 0.024), hypoxemia (OR 3.32; CI 1.23–8.93; p = 0.017), and severe undernutrition (OR 3.42; CI 1.29–9.12; p = 0.014).

**Conclusions and Significance:**

Non-breast feeding or cessation of breast feeding during the neonatal period may substantially increase the incidence of severe malnutrition, incidence of cough, and both the incidence and duration of hypoxemia in young infants presenting with pneumonia and diarrhea. The findings emphasize the paramount importance of the continuation of breast feeding in the neonatal period and early infancy.

## Introduction

Pneumonia and diarrhea are the two leading causes of death and morbidity in under-five children in developing countries [1], [2]. Among the 8.8 million global deaths in children under five in 2008, 18% and 15% occurred from pneumonia and diarrhea, respectively [1]. The death rate is even higher when children with pneumonia also present with hypoxemia [3], [4]. An increased incidence of breastfeeding is one of the important protective factors known to reduce deaths from pneumonia and diarrhea – the target is to reduce the under-five childhood mortality by two-thirds between 1990 to 2015 for achieving the MDG-4 [5], [6]. Despite attention and prioritization of improving the rate of breastfeeding, this remains one of the under achieved global targets for both developed and developing countries [7], [8], [9]. Continuation of breast-feeding at least during the first 6 months of life is important for adequate immunity against infection [10], [11]. Cessation of breast feeding within this period, especially during the neonatal period, is associated with a number of illnesses including diarrhea [12], pneumonia [13], [14], [15], and malnutrition [16]. Hypoxemia is one of the most dangerous sequels and an important risk factor of deaths from pneumonia [3], [4]. Management of hypoxemia requires adequate supplementation of oxygen which is often not accessible in resource limited settings [4]. Nevertheless, the need for oxygen therapy could be reduced by reducing the overall incidence of pneumonia. A recent study reported that young infants suckling on breast milk were better able to maintain oxygenation but desaturation occurred when they were not suckling [17]. We failed to identify any published literature showing the impact of cessation of breastfeeding during neonatal period on the development of hypoxemia in early infancy. A large number of young infants attend the Dhaka Hospital of International Centre for Diarrhoeal Disease Research, Bangladesh (ICDDR,B) with diarrhea each year, and some of them also have pneumonia and hypoxemia. The aim of our study was to assess the influence of the lack of breast feeding during the neonatal period on the subsequent development of clinical features of pneumonia, and hypoxemia in early infancy.

## Materials and Methods

### Patient enrollment

Infants of both genders, aged 0–6 months, admitted to the Special Care Ward (SCW) of the Dhaka Hospital of ICDDR,B with diarrhea and pneumonia from September through December 2007 were included into the study. Each year, this hospital provides care and treatment to over 120,000 patients of all ages. The majority of the patients come from a poor socio-economic background living in urban and peri-urban Dhaka. This being mainly a diarrhea treatment facility, essentially all patients have diarrhea with or without associated complications, but some may have other health problems. The majority of the patients are under-five children and malnutrition and pneumonia are the most common co-morbidities. On arrival to the hospital triage nurses obtain the medical history and make a quick assessment of the patients, focusing on the severity and complications of diarrhea and dehydration but also look for associated health problems. Following this, patients are referred either to the emergency physician for re-assessment or are admitted to an appropriate ward of the hospital. Patients with severe illnesses, including those with abnormal mental status, severe and very severe pneumonia, hypoxemia, cyanosis, suspected sepsis, and convulsions are admitted to the SCW for further assessment, closer observation, and appropriate laboratory workup and management. After admission to the SCW, attending physicians re-evaluate the patients, commence required work ups, and prescribe a management plan.

Originally this data has been obtained from a prospective hospital audit which was initially designed to defend the thesis in Masters of Medicine (MMed) of the primary author in the University of Melbourne (UOM), Melbourne, Australia. Although the clinical audit has been done prior to the approval by the Ethical Review Committee (ERC) of ICDDR,B because that was the routine clinical audit to assess the quality of hospital care, finally approval was obtained from the ERC after the completion of the clinical audit to defend the thesis of the primary author under the UOM.

### Study design

In this study, we prospectively enrolled all the diarrheal infants aged 0 to 6 months of both genders, who were admitted to the SCW with clinical and radiological evidence of pneumonia along with diarrhea from September 2007 through December 2007. Comparison of the clinical features of pneumonia, hypoxemia, and malnutrition was made between non-breast-fed and breast-fed infants. Non-breast-fed was defined as an infant who was never breast-fed or if breast feeding was stopped during the neonatal period. Breast-fed was defined as an infant who was exclusively or partially breast-fed from birth until the admission to the hospital. Pneumonia was clinically diagnosed according to the WHO criteria [18] and subsequently confirmed by radiological evidence of consolidation or patchy opacities. Hypoxemia was defined if the arterial oxygen saturation (SpO_2_) was below 90% according to the recommendation by WHO [19]. Diarrhea was defined as the passage of three or more abnormally loose or watery stool in the previous 24 hours. Relevant clinical information was collected soon after their enrollment into the study, after confirmation of oral consents by the attending physician from the parents/care-givers.

Standard guidelines of the hospital were followed in the clinical management of the study patients, which included correction of dehydration using either oral rehydration salt solution (for those with some dehydration) or intravenous fluids (for those with severe dehydration and also those who were unable to drink due to any reason); appropriate antimicrobial therapy; appropriate feeding, micronutrients, vitamins and minerals as and when indicated. Management of severe protein-energy malnutrition was done in accordance with the hospital's protocolised guidelines [20], [21].

### Statistical Methods

We developed case report forms (CRF), and pretested and finalized them for data acquisition. All data were entered onto a personal computer and edited before analysis using SPSS for Windows (version 15.0; SPSS Inc, Chicago) and Epi Info (version 6.0, USD, Stone Mountain, GA). Differences in proportions were compared by the Chi-square test or Fisher Exact test and differences of means were compared by Student's t-test or Mann-Whitney test, as appropriate. A probability of less than 0.05 was considered statistically significant. Strength of association was determined by a calculating odds ratio (OR) and its 95% confidence intervals (CI). We have these statistics both in our univariate analyses and logistic regression. Characteristics analyzed include age, weight, severe undernutrition [z score for weight for age <−3 of the median of the WHO anthropometry], distance from the hospital (km), cough with duration, fever with duration, inability to drink and its duration, hypoxemia with duration, respiratory rate (counted in one minute in a calm child), lower chest wall indrawing (indrawing of the bony structures of the lower chest wall during inspiration), head nodding, cyanosis, grunting respiration, and outcome. Initially, we performed univariate analyses of these characteristics to identify factors that were significantly associated with non-breast feeding and finally, we performed logistic regression analysis of the factors significantly associated with non-breast feeding after adjusting with potential confounders to identify the actual impact of non-breast feeding on clinical features of pneumonia and hypoxemia.

## Results

In total 107 infants were enrolled in the study of whom 73 were breast-fed and 34 were non-breast fed. The age (months) (mean ± standard deviation) and weight (kg) (mean ± standard deviation) of the breast-fed and non-breast fed infants were 2.9±1.5, 3.9±1.4 and 3.4±1.7, 3.6±1.3 respectively. All hypoxemic infants received oxygen therapy. The median (inter quartile) duration of hypoxemia (hours) in non-breast fed infants was longer compared to those in breast-feds [12.0 (0.0, 21.75) vs. 0.0 (0.0, 12.0); p = 0.021] (Table 1). The lowest SpO_2_ recorded at admission for the non-breast-fed and breast-fed infants were 63% and 70% on air respectively. After adjusting potential confounders, the non-breast fed infants had a higher incidence of cough, hypoxemia, and severe undernutrition (Table 2). The proportion of infants having a fatal outcome was higher among the non-breast fed infants but the difference was not statistically significant (Table 1). Parents with poor socio-economic status, illiteracy and age of mother, number of siblings and presence of smoker who smokes inside the house were comparable in both the groups (Table 3). Moreover, there were no significant differences in relation to distance from the hospital (km), fever (≥38° Celsius), duration of fever prior to admission, duration of cough prior to admission, inability to drink, duration of inabillity to drink prior to admission, respiratory rate, lower chest wall indrawing, head nodding, grunting respiration, cyanosis, and duration of hospitalization among the non-breast fed and breast-fed infants (Tables 1 & 2).

**Table 1:** Comparison of characteristics of breast-fed and non-breast-fed infants with pneumonia and diarrhea.

Characteristic	Non-breast-fed (n = 34)	Breast-fed (n = 73)	P
Age (months) (mean ± SD)	3.4±1.7	2.9±1.5	0.124
Weight (kg) (mean ± SD)	3.6±1.3	3.9±1.4	0.322
Distance from the hospital (km) [Median (IQR)]	18.0 (8.0, 25.0)	10.0 (8.0, 23.75)	0.331
H/o duration (hours) of fever at admission [Median (IQR)]	48.0 (21.0, 96.0)	72.0 (42.0, 120.0)	0.187
H/o duration (hours) of cough at admission; [Median (IQR)]	72.0 (36.0, 96.0)	72.0 (27.0, 144.0)	0.615
H/o duration (hours) inability to drink at admission; [Median (IQR)]	0.5 (0.0, 48.0)	0.0 (0.0, 24.0)	0.508
Respiratory rate (mean ± SD)	66±26	59±14	0.194
Total duration (hours) of hypoxemia (SPO2<90%) from admission [Median (IQR)]	12.0 (0.0, 21.75)	0.0 (0.0, 12.0)	0.021
Duration of hospitalization in days (mean ± SD)	4.7±3.7	5.4±3.2	0.254
Death (%)	4 (12)	4 (6)	2.3 (0.4–11.9)*0.261

IQR = inter quartile range, SD = standard deviation,

*Odds ratio (Confidence interval).

**Table 2:** Impact of breast feeding on clinical signs of pneumonia and hypoxemia in children also having diarrhea.

Variables	Non-breast-fed (n = 34) n (%)	breastfed (n = 73) n (%)	Non-adjusted		Adjusted	
			OR (95% CI)	P	OR (95% CI)	P
Fever (≥38°C)	26 (77)	63 (86)	0.52 (0.16–1.64)	0.323	0.41 (0.12–1.48)	0.175
Cough	32 (94)	60 (82)	3.47 (0.68–23.77)	0.136	9.09 (1.34–61.71)	0.024
Inability to drink	15 (44)	33 (45)	0.96 (0.36–2.35)	0.918	0.95 (0.38–2.39)	0.917
Lower chest wall indrawing	28 (82)	60 (82)	1.01 (0.31–3.36)	0.802	0.64 (0.16–2.55)	0.521
Head nodding	1 (3)	4 (6)	0.52 (0.02–5.31)	1.00	0.51 (0.05–5.72)	0.585
Grunting respiration	3 (9)	5 (7)	1.32 (0.23–6.91)	0.707	2.95 (0.42–20.46)	0.275
Cyanosis	6 (18)	8 (11)	1.74 (0.48–6.24)	0.366	2.04 (0.46–9.01)	0.347
Hypoxemia	25 (74)	36 (49)	2.85 (1.08–7.68)	0.032	3.32 (1.23–8.93)	0.017
Undernutrition (weigh for age <−3 z score)	25 (74)	35/71 (49)	2.86 (1.08–7.71)	0.033	3.42 (1.29–9.12)	0.014

Figure represents n (%), unless specified otherwise; OR = Odds ratio; CI = Confidence interval.

**Table 3:** Comparison of parental socioeconomic status and environment of breast-fed and non-breast-fed infants with pneumonia and diarrhea.

Variables	Non-breast-fed (n = 34)	Breast-fed (n = 73)	OR (95% CI)	p
Poor socio-economic status [(monthly income <5000 taka (US$ 67)]	20 (61)	42 (58)	1.10 (0.44–2.77)	0.995
Illiterate mother	15 (45)	33 (46)	0.98 (0.40–2.44)	0.861
Employed mother	6 (18)	12 (16)	0.76 (0.22–2.54)	0.824
Smoker in the family (inside the bed room)	15 (45)	42 (58)	0.60 (0.24–1.48)	0.308
Age of the mother in years (mean ± SD)	24.6±5.8	24.2±5.0	-	0.750
Number of siblings (mean ± SD)	1.5±0.7	1.4±0.7	-	0.948

Figure represents n (%), unless specified otherwise.

## Discussion

We observed a higher incidence of hypoxemia among non-breast-fed infants than breast-fed infants presenting with pneumonia and diarrhea. It is a novel and interesting finding for which we do not have a ready explanation. However, we may offer a number of potential explanations. Cessation of breast feeding in the neonatal period or non-breast feeding since birth is strongly associated with pneumonia [13]. Not all of our study infants with pneumonia were hypoxemic. Nonetheless, hypoxemia is one of the common sequels of severe pneumonia [4]. Non-breast-fed infants often have reduced immunity resulting from prolonged deprivation of highly immunogenic breast milk [22]. Breast milk contains certain factors, especially transforming growth factor (TGF)-β1 that is related to production of elastin [23], [24] which is needed for normal structural and functional development of the lungs [10], [25]. It is possible that non-breast-fed infants in our study had under developed and less well functioning lungs and pneumonia caused further deterioration of lung function leading to ventilation perfusion mismatch, the end result being hypoxemia. Obstructive sleep apnea-related hypoxemia that has a strong association with cessation of breast milk in neonatal period [26] could not be ruled out as one of the potential contributing factors for our observation as we did not have any data on that. The lowest observed SpO_2_ at admission was recorded in a non-breast-fed infant and non-breast-fed infants in our study more often remained hypoxemic than breast fed infants after O_2_ supplementation, which could also be due to their under developed and poorly functioning lungs. Ours was an observational study, and a well designed mechanistic future study is likely to provide an explanation for our findings.

The non-breast-fed infants more often presented with severe undernutrition. Anorexia in diarrheal neonates may reduce energy intake by 5 to 40% [27]; however, breast feeding usually remains unabated and intake may even increase during diarrhea [27], [28]. Therefore, lack of breast feeding during diarrhea further impacts nutrient and energy intake [27], [28]. However, the strong association of severe malnutrition with the cessation of breast feeding in early infancy has also been reported earlier [16]. The observation of a higher incidence of cough among our non-breast-fed study infants potentially signifies the protective effect of breast feeding on cough which has also been reported previously [29].

The case fatality was higher among non-breast-fed infants, although statistically insignificant. A number of previous studies have revealed a significantly higher death rate among non-breast-fed infants compared to those who were breast-fed [30], [31], and failure to attain statistical significance in our study is likely due to the small sample size, as reflected in wide 95% CI. Our observation of indifferent distribution of parental socioeconomic and environmental status among the non-breast-fed and breast-fed infants might also be due to same reason.

The main limitation of the study is the small sample which prevents further gain of statistical significant difference among the groups.

In conclusion, our data suggest that non-breast feeding or cessation of breast feeding during the neonatal period may substantially increase the incidence and duration of hypoxemia in association with pneumonia, as well as the incidence of cough and severe malnutrition in young infants presenting also with diarrhea. Our study identified as a new danger: the lack of breast feeding, or its discontinuation in the neonatal period, further highlighting the need for effective interventions to improve the rate of breast feeding and its continuation. A community-based prospective study with an adequate sample size is required to strengthen our observations.
